# Critical Thinking and Mental Health Nurse Education

**DOI:** 10.1111/inm.70021

**Published:** 2025-03-26

**Authors:** Jane Fisher

**Affiliations:** ^1^ School of Nursing and Midwifery University of Central Lancashire Preston UK

**Keywords:** critical thinking, criticality, genericism, mental health nurse education, mental health nursing

## Abstract

This paper examines critical thinking as a fundamental proficiency essential for inclusion in international mental health nurse education. Pragmatic solutions ensure the ongoing development of critical thinking in mental health nurses. The suitability of current mental health nurse education is under scrutiny globally. Critics claim that regulatory and educational standards have shifted from a focus on mental health‐specific skills to generic physical health‐based competencies, which do not adequately equip mental health nurses for clinical practice. The vital skill of critical thinking within mental health nurse theory and practice has been diluted (stating that it is a position paper or critical review, for example). This paper is a critical review of mental health nursing education. By summarising the global contextual background of generic‐based nursing education, it highlights the impact of the loss of mental health‐specific skill sets. Critical thinking is identified as a vital skill for the 21st‐century mental health nurse. This paper provides pragmatic suggestions to include critical thinking in nurse education in the absence of global reform and systemic educational change. Personal lived experience is used to illustrate the importance of critical thinking and service users’ experience of care. Critical thinking can be a partial solution to the current dilution of mental health nurse education occurring across the global North. Nurse educators should strive to embed critical thinking into mental health nurse education in order to develop lifelong critical thinkers who are not afraid to question the hegemonic perspective and continually ask, “why?” Recommendations are for nurse educators to be consciously aware of methods to encourage critical thinking, such as Socratic questioning, the consideration of epistemic injustice, coproduction, critical reflexivity and including case‐based ethical learning.

## 1 Introduction

This paper will examine critical thinking as a fundamental proficiency essential for inclusion in international mental health nurse education. The author of this paper occupies a dual position as a mental health lecturer and mental health service user, with first‐hand experience of receiving care from nurses appearing to lack criticality in their approach to service users. Critical thinking is offered as a partial solution to the current dilution of mental health nurse education occurring across the global North. With concerns around the progressive genericism of mental health nursing, this paper focusses on solutions within the current constraints of nurse education. Recommendations are for nurse educators to be consciously aware of methods to encourage critical thinking, such as Socratic questioning, the consideration of epistemic injustice, critical reflexivity and including case‐based ethical learning. Nurse educators should strive to embed critical thinking into mental health nurse education and develop lifelong critical thinkers who are not afraid to question the hegemonic perspective and continually ask, ‘why?’

## 2 Critical Thinking

Scholars find it challenging to define critical thinking, and the literature explores various pedagogical approaches to develop it. Definitions often include it being a cognitive process (Papathanasiou et al. 2014) and a set of learnable skills. It involves self‐awareness, reflexivity and the synthesis of new learning (Baker 1996). Within UK nurse education, critical thinking, analysis and reflection feature in the NMC Future Nurse Standards 1.7, 1.8, 6.8 (Nursing and Midwifery Council 2018), which outline competencies nurses should possess upon registration. Other international regulatory nursing bodies adopt similar tenets. Therefore, critical thinking forms a component part of international mental health nurse education. However, despite its standing, critical thinking is difficult to fully integrate into education without adopting a one‐dimensional or reductionist approach. Within the literature, there is no agreement on how best to teach or nurture critical thinking, or how to measure and assess it. This poses a problem for educators.

## 3 The Current Picture

There is a growing body of literature expressing grave concerns about global mental health nurse education. These centre on the dilution of mental health‐specific skills and a progressive move towards the genericism of nurse education. The global trend for nurse education largely focusses on a generic curriculum, with students specialising in mental health post registration. Australia adopts such a model yet has been extensively criticised for failing to prepare nurses to work in mental health settings (Hurley and Ramsay 2008). Among other critics, Lakeman et al. (2023) advocate for a return to preregistration specialist training.

In contrast to this generic model, the United Kingdom permits student nurses to select one of the four fields of nursing (adult, mental health, children and young people or learning disability) to study at preregistration level. However, there are growing concerns over the ability of this apparent specialised curriculum to adequately equip students for their specialised chosen fields. Based on fervent critique of the generic undergraduate education of nurses in Australia, UK policymakers were alerted to the risks of losing mental health‐specific skills. However, many critics (Bifarin et al. 2024; Warrender et al. 2023) believe this warning was ignored in the updated Future Nurse: Standards of Proficiency for Registered Nurses (Nursing and Midwifery Council 2018).

Connell et al. (2022) contend the updated UK NMC standards represented a shift away from person‐centred care and a mental health nursing approach, towards an educational model constructed on quantifiable skills and competencies. Critics argue this focus on physical health‐based competencies best suited to adult nurses has meant the (unintended) reduction and dilution of mental health‐specific skills, including criticality (Warrender et al. 2023). It is important to examine the context of this UK philosophical shift, as it sits within a backdrop of momentous physical healthcare scandals that should never be repeated. The Mid Staffordshire hospitals severe failings in patient care triggered a review of preregistration nurse education. The Willis Report (Willis 2015) prioritised the physical health needs of the population and recommended that nurse education employ a 2‐year shared content and one‐year field‐specific content. Despite this never being made mandatory by the UK nursing regulator, it was widely adopted by UK Higher Education Institutions (HEI's).

This philosophical move towards the genericism of the nursing curriculum is a concern for mental health nurse educators. Teaching and learning activities are affected, alongside the preparedness for practice (or satisfaction) that students feel at the end of their educational programme. Teaching time is consumed on physical health interventions such as cannulation and catheterisation, which mental health nurses are unlikely to use in clinical practice. Mental health‐specific content is desperately shoehorned into generic shared modules, compromising the quality of such content.

Insufficient time allocated to specialised content results in the dilution of mental health‐specific skills. Pachkowski (2018) acknowledges that mental health nurse educators are lamenting the demise of a comprehensive critical ethics education. Because of the nature of navigating moral and ethical issues and a contested evidence base for hegemonic interventions, mental health nurses are required to make and justify complex decisions daily. They frequently negotiate ethical tension and values conflict, which needs to be reflected in their curriculum content. Legislation and the legal removal of human rights heighten power differentials. Mental health nurses are positioned to offer care and control simultaneously, which requires a highly specialised education, different from our dominant adult counterparts.

Service users (such as I) are seeking genuine human connection amidst severe mental distress and temporary loss of agency and control (Fisher 2023b). To meet these needs effectively, mental health nurses need to be reflexive, analytical, critical thinkers. This must be embedded and nurtured within nurse education to meet service users' needs. Without adequate curriculum content that addresses the unique and challenging role of a mental health nurse, standards of care may decrease (Warrender et al. 2023). As a mental health service user at the mercy of mental health services, this is a grave and sobering concern. When care is often lacking in meaningful connection and substance, the idea of care deteriorating further because of educational standards is both deeply disturbing and incites a visceral fear of substandard or even negligent care. The psychiatric survivor movement has a long and fervent history of accusing mental health nurses of being paternalistic and coercive (Aves 2024). Is it possible this could get worse?

Literature to date on the dilution and genericism of mental health nurse education has made impassioned calls for allies to unite (McKeown 2023) and mental health nursing to rise from the ashes (Warrender et al. 2023). The NMC has been petitioned by open letters from the grassroots movement ‘Mental Health Deserves Better’ to urgently review standards for education (Mental Health Deserves Better 2023). Whilst in agreement with these bold calls to action, this paper focusses on day‐to‐day pragmatic solutions for educators on the front line of mental health nurse education. Until global, institutional and regulatory constraints can be removed or diminished, action on the front line, by both individuals and teams of mental health nurse lecturers, is vital. Service users who are at the mercy of mental health nurses deserve a reform in educational standards and curriculum content.

After reviewing the failings of a generic nurse education, this paper will now explicate pragmatic solutions to the theory–practice gap and dilution of critical thinking as a mental health‐specific skill.

## 4 Potential Solutions

### 4.1 Integration of Critical Thinking Skills

The nursing curriculum should embed critical thinking throughout. However, this is challenging when one examines the plethora of skills and values competing for integrated inclusion in mental health nurse education. Alongside critical thinking is compassion, empathy, evidence‐based practice, reflection and other nursing values, all with a logical rationale for prioritisation. Cleary et al. (2023) advocate embedding and integrating critical thinking into mental health nurse education; it then becomes a skill in one's professional ‘toolkit’. We can teach critical thinking explicitly as a skill (Andreucci‐Annunziata et al. 2023) facilitated by library and academic skills facilitators. If these principles are followed up in seminars, students can explore the applicability to mental health nursing clinical practice. It is vital students see critical thinking as a core skill required for both academic and clinical work. This will create lifelong critical thinkers, comparable with the aims of heutagogy. As an extension to andragogy, heutagogy has been heralded as the preferred pedagogical approach to higher education teaching and learning (Kenyon and Hase 2001). It aims to develop independent, lifelong and self‐motivated learners.

### 4.2 Case‐Based Group Work

According to Pachkowski (2018), case‐based group work is an effective way to embed ethics and critical thinking into mental health nurse education. Case‐based group work partially addresses the need for criticality in mental health nurse education. Group work facilitates learning as students make sense of things together, examine and discuss concepts, and make collective decisions. This mirrors the requirements of a registered nurse in clinical practice (Nursing and Midwifery Council 2018) whilst aligning with principles of heutagogy (Kenyon and Hase 2001). Case‐based ethics sessions can support the development of self‐directed, motivated and independent learners. This is of particular significance to year three students with their nursing registration imminent.

### 4.3 Socratic Questioning

Among international calls for technology‐enabled learning and active blended learning, this paper implores mental health lecturers to not abandon techniques such as Socratic questioning in teaching and learning activities. The ancient Socratic method of teaching fosters critical enquiry to challenge preconceived thoughts and established beliefs in line with critical thinking (Bates 2023). It encourages students to examine the reasons they do what they do. This leads to greater confidence in being able to justify their nursing decision‐making amidst complex ethical dilemmas. A key to this is creating situations where students feel safe questioning and reflecting on their own experiences and values. Socratic pedagogy is an engaging teaching strategy, which encourages students to be active participators in their own learning. This again aligns with the pedagogical principles of heutagogy (Kenyon and Hase 2001).

### 4.4 Critical Reflection

A vital part of critical thinking is critical reflection on students' own values, biases, beliefs and assumptions. Regulatory bodies, such as the Nursing and Midwifery Council in the United Kingdom, enshrine this critical reflexivity within their requirements. Critical thinking is a practice of transforming one's relationship with oneself (Foucault 2007) Cleary et al. (2023) recommend that critical thinking begins when students reflect on themselves and what shapes their nursing practice. This can be uncomfortable for students and requires skilled facilitation. It is, however, necessary for critical reflexivity.

In my teaching practice, I have facilitated challenging reflexive discussions on sociocultural and political views or assumptions within generic sessions on health inequalities. With careful reminders of sensitivity and respect, students will also question and challenge each other. This could lead to a sudden escalation of chaos, particularly if students express less than desirable values. In these situations, it is crucial to address values or attitudes in opposition to nursing values. I have witnessed important self‐realisation and shifts in views when students find themselves challenged by their peers. If needed, this can also be followed up on a one‐to‐one basis.

### 4.5 Epistemic Injustice

I have previously argued for the inclusion of epistemic injustice in mental health nurse education (Fisher 2024). As a philosophical theory relating to injustices that occur around knowledge exchange and interpretation (Fricker 2007), it can be used as a framework to understand the dismissal and silencing of mental health service users (Fisher 2024; Kidd et al. 2022). Despite potentially being an unfamiliar term to student mental health nurses, the prevalence of the phenomena is easily identifiable in the dismissing and doubting of service user's testimony and individual interpretations. Epistemic injustice provides a critical lens to view and examine service users' experiences of care, and how mental health nurses and students can perpetuate epistemic injustices. This deep critical reflexivity encompasses ethical issues pertaining to power asymmetries, social inequalities and stigma.

I have previously outlined a practical application of epistemic injustice to mental health nurse education to provoke critical thinking and reflexivity (Fisher 2024). This includes honouring the voice of the service user without seeking to rewrite or reinterpret someone's experiences (Buxton 2023). Exploring critical literature with students, for example Asylum magazine (www.asylummagazine.org), and Mad Studies (Beresford and Russo 2022) encourages students to consider different perspectives of mental health care and their potential collusion with epistemically unjust practices.

## 5 Coproduction

The value of lived experience and learning from service users as an effective teaching tool is well established in pedagogical literature. However, there is a risk that only a few service users with social and educational status are permitted into the university classroom. This represents elite capture (Okoroji et al. 2023) and a challenge for educators wishing to foster critical thinking in nursing students. Educators must ensure they do not perpetuate epistemic injustice by heavily sanitising people's lived experience or cherry‐picking inspirational stories of recovery and resilience. In order to foster critical thinking in students, they need to be exposed to the reality of iatrogenic harm (Aves 2024), the toxic neoliberal undertones of resilience (Fisher and Jones 2023) and a range of nuanced personal narratives. If lived experience involvement is limited to an articulate elite capturing celebrating mainstream mental health services, any critical thinking outside of this narrative is suppressed.

## 6 Assessing Criticality

Assessing criticality is challenging (Andreucci‐Annunziata et al. 2023). Module learning outcomes require critical and analytical exploration, discussion and synthesis, usually assessed as a written assignment. There are strengths and limitations to this assessment method. University policy on grading assignments and marking rubrics can in themselves stifle critical thinking. Whilst they can guide students in their academic writing by providing explicit criteria for critical thinking, they can also turn critical writing into a logical science. However, mental health nursing is both an art and a science (Chambers 2017). Adam and Juergensen (2019) argue that regulated and directed critical thinking eclipses artistic and creative expression. This can remove the heart of critical thinking, or what Foucault (2007) describes as a virtue and ethical self‐transformation. Is it possible to accurately grade this against an assessment rubric?

This paper urges mental health nurse educators to continually question the fundamental assumptions of the hegemonic frameworks within psychiatry. To teach critical thinking, educators need to model critical thinking. Cleary et al. (2023) argue that nurse education is heavily influenced by the language and viewpoint of psychiatry. Adam and Juergensen (2019) contend that the hierarchy of teaching material eclipses critical thought. At the top of the hierarchy are foundational nursing textbooks that dictate curriculum content. However, a growing body of evidence exists, for example (Fisher 2023a; Russo and Beresford 2015; Davies 2021; Sidley 2015), that adopts a critical approach to the psychiatric discourse. These critical voices question the legitimacy of mental illness and diagnosis as a distinctive entity, plus psychiatry's authority over human emotions and behaviour.

## 7 Biomedical Frameworks

Mental health nurse education often adopts an overwhelming biomedical standpoint (Pachkowski 2018) where mental illness sits within a medical framework. This represents a curriculum colonised by psychiatry and limits critical thinking outside of this model. In a Canadian institutional ethnography, Adam (2017) found an institutional and discursive dominance of biomedical psychiatry. This paper contends that there are many mental health nurse lecturers who hold a diverse and eclectic view of psychiatry and mental distress. Despite them wishing to foster critical thinking within students, there still are institutional, regulatory and societal constraints that make this problematic.

At the end of their education, students will graduate and gain employment as a registered mental health nurses in a system that is founded on biomedical influences. If there is too much focus on critical thinking for critical thinking's sake, students will be unequipped to enter the contemporary workforce. There is already a theory to practice gap, and a tendency for psychiatric debates to collapse into unhelpful binaries (McKeown 2023). A focus on alternative perspectives at the expense of biomedical approaches to mental distress could exacerbate this gap. There is also a high risk that new nurses are dismissed as idealistic and forced to conform to the dominant existing culture of nursing. This is provocatively referred to as nurses eating their young (Meissner 1986) and remains a contemporary concern (Warrender 2022) An obvious common‐sense balance is required. Students need an education to equip them to enter the contemporary nursing workforce. Simultaneously, they need to be aware of the limitations of mainstream psychiatry, with the ability and confidence to continuously ask ‘why?’ This is the crux of critical thinking.

## 8 A Personal Perspective

As a mental health service user living within an imposed category of severe and enduring mental illness, I am often at the mercy of the mental health system. My personal narrative was rewritten and reinterpreted through the lens of biomedical psychiatry. I have experienced care ranging from adequate to entirely substandard and poor. However, there are occasional glimpses of a mental health professional who dares to ask a tender and curious ‘why?’ Closely followed with ‘what is best for this person in front of me?’ Their gentle professional curiosity incites individual meaning making and truly personalised care. The mental health system is shaped to meet my needs, as opposed to being forced into a rigid system of reductionism and one‐dimensional flow charts that arguably fit no one. My experiences and personal narrative remain my own, safe from reinterpretation by a pathologising disease approach to mental distress. This requires dynamic critical thinking clinicians who are prepared to go against the status quo and genuinely advocate for service users.

## 9 The Future of Mental Health Nursing

The status of ‘psychiatric nurse’ itself is posited as outdated and untenable in the light of damning evidence against biomedical interventions (Wand 2024). Does the future of mental health nursing include conscientious objection to enforced pharmaceutical interventions? (Gadsby and McKeown 2021) Should mental health nursing break free from the title of nurse and develop a new professional identity? These are the challenges and dilemmas that the future workforce and academics will grapple with. It is our duty to shape and nurture future nurse leaders and academics. If they can learn from the mistakes of the past, maybe a paradigm shift can occur in mental health care. This is impossible to achieve without valuing the skill of critical thinking in nurse education.

## 10 Conclusion

In conclusion, there is no dispute about the importance of critical thinking in the literature; however, the pedagogical approaches are varied. Regulatory standards, generic modules, institutional frameworks around assessment and a biomedical hegemony existing within the culture of nurse education constrain mental health nurse lecturers and educators. As important as the radical claims for reform and uprising are, the day‐to‐day efforts of educators to be mindful of nurturing critical thinking in mental health nursing students are of equal importance. Critical thinking students becoming critical thinking nurses, positioned within mental health services, with the courage to ask the gentle and sensitive ‘why?’

## Conflicts of Interest

The author declares no conflicts of interest.

## Data Availability

Data sharing not applicable to this article as no datasets were generated or analysed during the current study.

## References

[inm70021-bib-0001] Adam, S. 2017. “Crazy Making: The Institutional Relations of Undergraduate Nursing in the Reproduction of Biomedical Psychiatry.” International Journal of Nursing Education Scholarship 14, no. 1: 65. 10.1515/ijnes-2017-0071. 29240556

[inm70021-bib-0002] Adam, S. , and L. Juergensen . 2019. “Toward Critical Thinking as a Virtue: The Case of Mental Health Nursing Education.” Nurse Education in Practice 38: 138–144. 10.1016/j.nepr.2019.06.006. 31279924

[inm70021-bib-0003] Andreucci‐Annunziata, P. , A. Riedemann , and S. Cortés . 2023. “Conceptualizations and Instructional Strategies on Critical Thinking in Higher Education: A Systematic Review of Systematic Reviews.” Frontiers in Education 8: 1686.

[inm70021-bib-0004] Aves, W. 2024. “Escaping Iatrogenic Harm: A Journey Into Mental Health Service Avoidance.” Journal of Psychiatric and Mental Health Nursing 31, no. 4: 668–673. 10.1111/jpm.13020. 38234176

[inm70021-bib-0005] Baker, C. R. 1996. “Reflective Learning: A Teaching Strategy for Critical Thinking.” Journal of Nursing Education 35, no. 1: 22. 10.3928/0148-4834-19960101-06 8926510

[inm70021-bib-0006] Bates, B. 2023. Learning Theories Simplified: How to Apply Them to Teaching. Sage.

[inm70021-bib-0007] Beresford, S. , and B. Russo , eds. 2022. The Routledge International Handbook of Mad Studies. Routledge.

[inm70021-bib-0008] Bifarin, O. , F. Collier‐Sewell , G. Smith , et al. 2024. “Standards of Proficiency for Registered Nurses—To What End? A Critical Analysis of Contemporary Mental Health Nursing Within the United Kingdom Context.” Nursing Inquiry 31, no. 3: e12630. 10.1111/nin.12630. 38436620

[inm70021-bib-0009] Buxton, O. 2023. “Not My Words, Not My Story.” Asylum Magazine 30, no. 4: 433.

[inm70021-bib-0010] Chambers, M. 2017. Psychiatric and Mental Health Nursing: The Craft of Caring. Routledge.

[inm70021-bib-0011] Cleary, M. , S. West , and C. Hungerford . 2023. “Four Steps to Add Critical Thinking to the Mental Health Nursing Toolkit.” Issues in Mental Health Nursing ahead‐of‐print(−) 44, no. 11: 1167–1170. 10.1080/01612840.2023.2212813. 37319420

[inm70021-bib-0012] Connell, C. , E. Jones , M. Haslam , J. Firestone , G. Pope , and C. Thompson . 2022. “Mental Health Nursing Identity: A Critical Analysis of the UK's Nursing and Midwifery Council's Pre‐Registration Syllabus Change and Subsequent Move Towards Genericism.” Mental Health Review Journal 27, no. 4: 472–483. 10.1108/mhrj-02-2022-0012.

[inm70021-bib-0013] Davies, J. 2021. Sedated: How Modern Capitalism Created Our Mental Health Crisis. Atlantic Books.

[inm70021-bib-0014] Fisher, J. 2023a. “Epistemic Injustice: The Silenced Voices.” International Journal of Mental Health Nursing 32, no. 4: 1186–1189. 10.1111/inm.13163. 37170848

[inm70021-bib-0015] Fisher, J. 2023b. “The True Value of a Mental Health Nurse: The Lessons That I Learned by Becoming a Mental Health Service User.” British Journal of Mental Health Nursing 12, no. 1: 1–3.

[inm70021-bib-0016] Fisher, J. 2024. “Is Epistemic Injustice a Worthy Application to Mental Health Nurse Education?” Nursing Ethics 31, no. 7: 9697. 10.1177/09697330241259154. PMC11470644 39120121

[inm70021-bib-0017] Fisher, J. , and E. Jones . 2023. “The Problem With Resilience.” International Journal of Mental Health Nursing 33, no. 1: 185–188. 10.1111/inm.13220. 37665109

[inm70021-bib-0018] Foucault, M. 2007. The Politics of Truth. Semiotext(e).

[inm70021-bib-0037] Fricker . 2007. Epistemic injustice: Power and the ethics of knowing. Oxford University Press.

[inm70021-bib-0019] Gadsby, J. , and M. McKeown . 2021. “Mental Health Nursing and Conscientious Objection to Forced Pharmaceutical Intervention.” Nursing Philosophy 22, no. 4: nup.12369. 10.1111/nup.12369. 34463024

[inm70021-bib-0020] Hurley, J. , and M. Ramsay . 2008. “Mental Health Nursing: Sleepwalking Towards Oblivion?” Mental Health Practice 11, no. 10: 14–17.

[inm70021-bib-0021] Kenyon, C. , and S. Hase . 2001. “Moving from Andragogy to Heutagogy in Vocational Education.”

[inm70021-bib-0022] Kidd, I. J. , L. Spencer , and H. Carel . 2022. “Epistemic Injustice in Psychiatric Research and Practice.” Philosophical Psychology 38, no. 2: 1–29. 10.1080/09515089.2022.2156333.

[inm70021-bib-0023] Lakeman, R. , K. Foster , M. Hazelton , C. Roper , and J. Hurley . 2023. “Helpful Encounters With Mental Health Nurses in Australia: A Survey of Service Users and Their Supporters.” Journal of Psychiatric and Mental Health Nursing 30, no. 3: 515–525. 10.1111/jpm.12887. 36440476

[inm70021-bib-0024] McKeown, M. 2023. “Resisting Genericization: Towards the Renewal of Mental Health Nursing.” Journal of Advanced Nursing 79, no. 9: 3189–3191. 10.1111/jan.15598. 36797810

[inm70021-bib-0025] Meissner, B. 1986. “Nurses: Are We Eating Our Young?” Nursing 3, no. 16: 51–53. 10.1097/00152193-199902000-00018. 3633461

[inm70021-bib-0026] Mental Health Deserves Better . 2023. “Open Letter on Nurse Education.” Nursing Times. https://www.nursingtimes.net/opinion/open‐letter‐on‐nurse‐education‐mental‐health‐deserves‐better‐21‐02‐2023/.

[inm70021-bib-0027] Nursing and Midwifery Council . 2018. “The Code: Professional Standards of Practice and Behaviour for Nurses, Midwives and Nursing Associates.”

[inm70021-bib-0028] Okoroji, C. , T. Mackay , D. Robotham , D. Beckford , and V. Pinfold . 2023. “Epistemic Injustice and Mental Health Research: A Pragmatic Approach to Working With Lived Experience Expertise.” Frontiers in Psychiatry 14: 1114725. 10.3389/fpsyt.2023.1114725. 37056406 PMC10086175

[inm70021-bib-0029] Pachkowski, K. S. 2018. “Ethical Competence and Psychiatric and Mental Health Nursing Education. Why? What? How?” Journal of Psychiatric and Mental Health Nursing 25, no. 1: 60–66. 10.1111/jpm.12439. 29105208

[inm70021-bib-0030] Papathanasiou, I. V. , C. F. Kleisiaris , E. C. Fradelos , K. Kakou , and L. Kourkouta . 2014. “Critical Thinking: The Development of an Essential Skill for Nursing Students.” Acta Informatica Medica 22, no. 4: 283–286. 10.5455/aim.2014.22.283-286. 25395733 PMC4216424

[inm70021-bib-0031] Russo, J. , and P. Beresford . 2015. “Between Exclusion and Colonisation: Seeking a Place for Mad People's Knowledge in Academia.” Disability & Society 30, no. 1: 153–157. 10.1080/09687599.2014.957925.

[inm70021-bib-0032] Sidley, V . 2015. “Tales From the Madhouse: An Insider Critique of Psychiatric Services.”

[inm70021-bib-0033] Wand, T. 2024. “We Have to Cancel Psychiatric Nursing and Forge a New Way Forward.” International Journal of Mental Health Nursing 33, no. 2: 215–219. 10.1111/inm.13301. 38308416

[inm70021-bib-0034] Warrender, D. 2022. “Mental Health Nursing and the Theory‐Practice Gap: Civil War and Intellectual Self‐Injury.” Journal of Psychiatric and Mental Health Nursing 29, no. 2: 171–173. 10.1111/jpm.12808. 34860457

[inm70021-bib-0035] Warrender, D. , C. Connell , E. Jones , et al. 2023. “Mental Health Deserves Better: Resisting the Dilution of Specialist Pre‐Registration Mental Health Nurse Education in the United Kingdom.” International Journal of Mental Health Nursing 33, no. 1: 202–212. 10.1111/inm.13236. 37788130

[inm70021-bib-0036] Willis, S. 2015. “Raising the Bar Shape of Caring: A Review of the Future Education and Training of Registered Nurses and Care Assistants.” https://www.hee.nhs.uk/sites/default/files/documents/2348‐Shape‐of‐caring‐review‐FINAL.pdf.

